# Absence of Evidence for MHC–Dependent Mate Selection within HapMap Populations

**DOI:** 10.1371/journal.pgen.1000925

**Published:** 2010-04-29

**Authors:** Adnan Derti, Can Cenik, Peter Kraft, Frederick P. Roth

**Affiliations:** 1 Department of Biological Chemistry and Molecular Pharmacology, Harvard Medical School, Boston, Massachusetts, United States of America; 2 Department of Epidemiology, Harvard School of Public Health, Boston, Massachusetts, United States of America; 3 Department of Biostatistics, Harvard School of Public Health, Boston, Massachusetts, United States of America; University of Chicago, United States of America

## Abstract

The major histocompatibility complex (MHC) of immunity genes has been reported to influence mate choice in vertebrates, and a recent study presented genetic evidence for this effect in humans. Specifically, greater dissimilarity at the MHC locus was reported for European-American mates (parents in HapMap Phase 2 trios) than for non-mates. Here we show that the results depend on a few extreme data points, are not robust to conservative changes in the analysis procedure, and cannot be reproduced in an equivalent but independent set of European-American mates. Although some evidence suggests an avoidance of extreme MHC similarity between mates, rather than a preference for dissimilarity, limited sample sizes preclude a rigorous investigation. In summary, fine-scale molecular-genetic data do not conclusively support the hypothesis that mate selection in humans is influenced by the MHC locus.

## Author Summary

There is evidence in numerous species that genes involved in immunity influence mate choice. Factors like body odor may subconsciously favor partners with different immunity alleles, to avoid inbreeding and/or endow offspring with broad resistance to pathogens. A previous study, based on HapMap genotypes, reported that European-American mates were extremely dissimilar from each other in immunity alleles compared to non-mates. Upon re-examining the results and methods, and visually comparing mates and non-mates, we found that this effect was weak, strongly dependent on extreme pairs and on arbitrary choices in methodology, and not significant after correcting for the multiple hypotheses tested. More importantly, examination of new couples from the same population did not support this hypothesis. Rare instances of very high MHC similarity among non-mates suggest that mates may avoid extreme similarity, rather than favoring dissimilarity. However, too few samples are readily available to test this prospect rigorously. We conclude that HapMap samples do not conclusively support the hypothesis that MHC genotypes exert an influence on mate choice. The same previous study reported that Yorubans appear to prefer mates who are more genetically similar to themselves overall. Our analyses suggest that the effect is driven by a subset of the sample.

## Introduction

The MHC locus contains genes central to acquired immunity, as well as numerous olfactory receptors [1]. It is reported to influence mate selection in a number of vertebrates, and is thought to act through the sense of smell to favor genetic dissimilarity between parents and thus heterozygosity in offspring [2]. Evidence for these effects in humans includes the high degree of MHC polymorphism [1], MHC-dependent female sexual interest [3] and preferences for male body odors [4]–[6], and a depletion of matching five-locus HLA haplotypes in Hutterite couples [7]. Among relevant experiments in model organisms, MHC class I peptides were shown to induce pregnancy blocking in mice [8]. However, this block required the vomeronasal organ, which is not known to function in humans, and human pheromones have not been clearly identified [9].

Despite the advent of MHC-based matchmaking services [10], not all of the available evidence consistently supports a preference for MHC-dissimilar mates in humans [11]. Although women indicated a preference for the odors of MHC-dissimilar men when knowingly [4] or unknowingly [5] rating potential partners, women who were single [6] or taking oral contraceptives [4], [6] preferred the odors of MHC-similar men. Women also ranked the faces of MHC-similar men as more attractive [12]. In addition, the relationship of odor preference in these controlled settings to the selection of actual mates in practice is unclear.

HapMap genotypes of father-mother-child trios afford the chance to test for an association of MHC similarity with *bona fide* mate selection rather than a stated preference. A recent study by Chaix *et al.* sought signs of mate selection in the genotypes of HapMap Phase 2 (Hap2) parents by comparing the genetic relatedness of mated and unmated opposite-sex couples, both at the MHC locus and overall (using all common autosomal variants) [13]. Yoruban mates (N = 27 couples) were reported to be slightly more similar than expected (nominal two-sided *P*<0.001) overall, but no significant difference in MHC relatedness was detected between mates and non-mates. By contrast, European-American mates (N = 28 couples) did not differ significantly from non-mates in autosomal relatedness, but were less similar at the MHC locus than were non-mates (*P* = 0.015).

**Figure 1:**
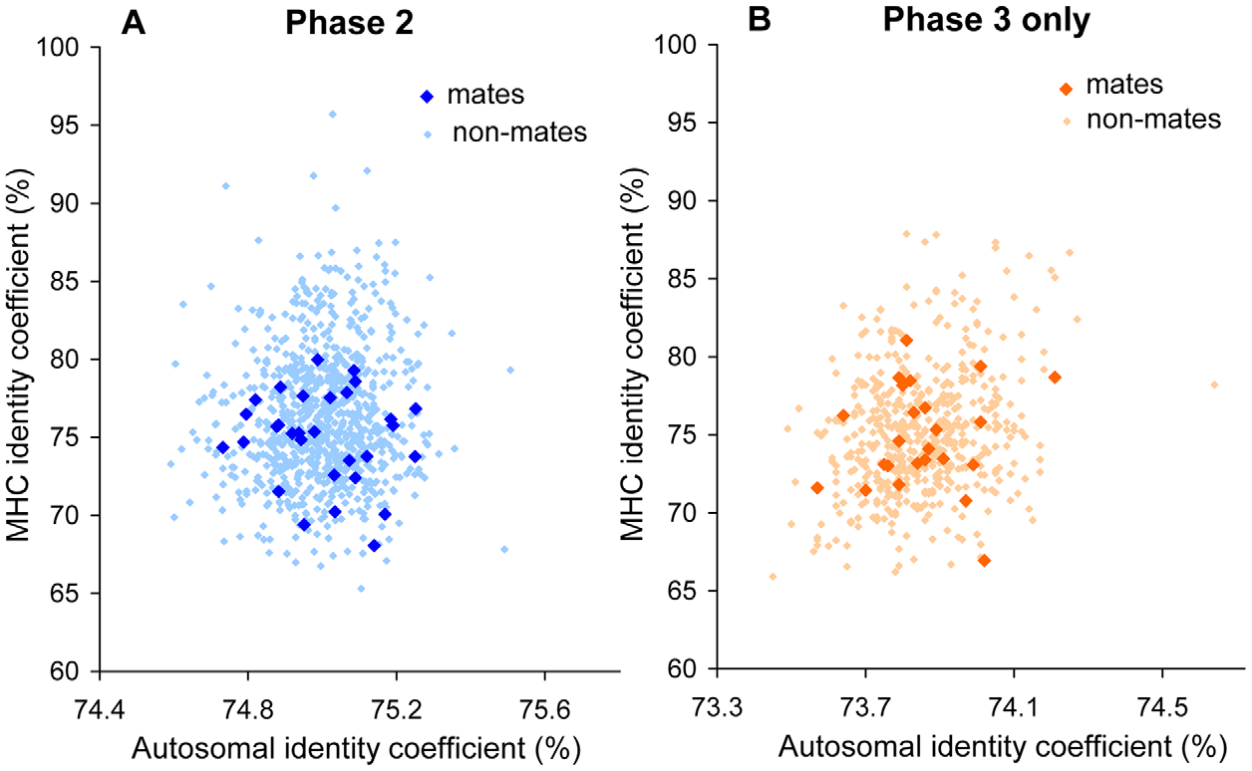
Identity coefficients of HapMap European couples. Autosomal and MHC identity coefficients are plotted for mate pairs and corresponding non-mate male-female pairs for (A) Phase 2 genotypes (30 couples) and (B) Phase 3 genotypes of individuals not included in Phase 2 (24 couples). Close relatives (see Text S2) are not shown. Coefficients were based on unphased genotypes, SNPs with MAF≥1%, and a het-het score of 1 (see Methods). Autosomal coefficients were lower in Phase 3 because fewer SNPs with low minor allele frequencies were genotyped. Same-sex coefficients are not shown but were included in calculations of relatedness.

**Figure 2:**
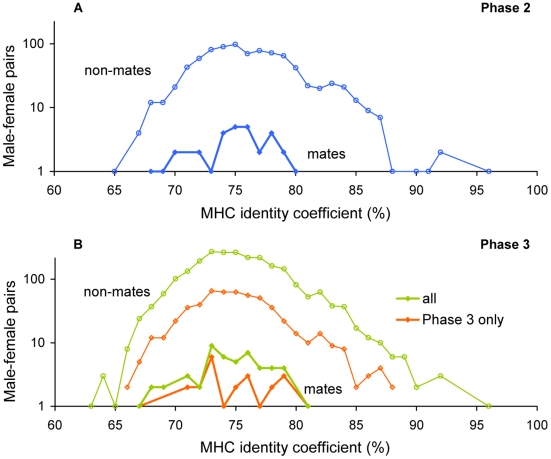
MHC identity in HapMap Europeans. Distributions of MHC identity coefficients are shown for mates and for non-mate male-female pairs in (A) Phase 2 (30 couples), and (B) Phase 3 (“all”; 50 couples), as well as the subset of Phase 3 couples not genotyped in Phase 2 (“Phase 3 only”; 24 couples). Close relatives were excluded.

The latter result was interpreted as supporting a role for the MHC locus in mate choice, and outliers were excluded as an explanation [13]. However, a visual comparison of mate and non-mate pairs (Figure 1 and Figure 2) suggests a weak effect that may derive from a few extreme pairs. Furthermore, adjusting the significance threshold for the fact that multiple hypotheses were tested (two sets of SNPs in each of two populations) would have rendered the results insignificant (see Results).

The availability of HapMap Phase 3 (Hap3) data now permits a test of these findings using independent samples from the same population.

## Methods

### Replication of previous results

The findings reported previously in Hap2 couples [13] were replicated as follows. Phased genotypes (release 21) for Yorubans and Europeans were obtained from the HapMap web site (http://www.hapmap.org). There were 30 mated couples in each cohort (see Text S1 for a description of samples and data sets). Only autosomal SNPs were retained. The same couples were excluded as in the previous study to avoid the presence of close relatives (Text S2), based on independent calculations of relatedness coefficients [14]. In each population, SNPs with a minor allele frequency (MAF) <5% (based on remaining samples) were excluded. For every pair of individuals *a* and *b*, the identity coefficient, *Q_a,b_*, was calculated as the fraction of identical alleles, but with two heterozygotes considered 50% identical (het-het  = 50%). While Chaix *et al.* referred to *Q* as the proportion of identical variants, a comparison of an individual with themselves (self-self) using the het-het  = 50% score yields values of *Q* that are variable and lower than unity (see Supporting Figure 1 in Text S3); we therefore refer to *Q* as an identity coefficient.

The mean *Q* for all pairs within the cohort (after excluding relatives), *Q_mean(cohort)_*, was then derived, and the relatedness coefficient for *a* and *b* was calculated as *R_a,b_*  =  (*Q_a,b_*−*Q_mean(cohort)_*)/(1−*Q_mean(cohort)_*). The mean *R* (*R_mean_*) for couples, *R_mean(mates)_*, was calculated; its significance was then assessed by randomly pairing males and females 1,000 times and recording the frequency *P* with which the absolute value of *R_mean_* from a random trial was equal to or greater than that observed for real couples. Following the previous report, actual couples were allowed in random trials; however, we found that excluding them had little impact on the results (data not shown; also see description of *Z* score below). *Q*, *R* and *P* values were also calculated based on SNPs at the MHC locus, defined as spanning positions 29,700,000 to 33,300,000 on chromosome 6 for NCBI build 35 [13]. Results are shown in Table S1.

### Modification of methods

The methods were modified in various ways, both to explore the robustness of results to changes in the procedure, and to establish the applicability of the analysis to Hap3 couples; details are provided in Text S3 and results are shown in Table S2 and Table S3. First, in order to facilitate permutations of excluded couples due to relatives (see below), we calculated MAFs before excluding couples, rather than after, and determined that this change had a negligible effect on results (Table S2 and Table S3). Second, in addition to *P* values, the difference in relatedness for mates and non-mates was quantified as a *Z* score, namely (*R_mean(mates)_*−*R_mean(non-mates)_)*/*R_sd(non-mates)_*, where *R_mean(non-mates)_* and *R_sd(non-mates)_* are the mean and standard deviation, respectively, of the relatedness of non-mate pairs. Because we wished to assess our results using the methods of the previous report [13], we compared means rather than medians, and based the *Z* score on standard deviations rather than median deviations. We note that *P* values are based on random trials in which real mate pairs were allowed, for consistency with the previous report [13], while *Z* scores compare mates with the non-mate distribution; however, this distinction has a negligible impact on results (data not shown).

We observed inconsistent *P* values derived from different sets of 1,000 random trials (Table 1). This result was to be expected based on the theoretical standard error, calculated as (.05*.95/1000)^1/2^ [15], or 14% of *α*, for empirical *P* values near *α* = 0.05. We therefore increased the number of random trials to 100,000, which reduced the standard error in empirical *P* values by a factor of 10. Because the results varied according to which couples were excluded, we opted to analyze all possible permutations and to calculate aggregate *R*, *P* and *Z* values. To enable this analysis, *Q_mean(cohort)_* and all pairwise *R* values were first calculated with all samples included in order to detect relatives (see Text S2 for details). Subsequently, for each permutation of excluded couples, *Q_mean_* and *R* values were recalculated based on remaining couples, followed by random trials.

**Table 1:** Summary of MHC relatedness analyses in HapMap Europeans.

	Methods			Mate pairs		Relatedness x10^3^		Significance		
Genotypes^1^	MAF	Het^2^	Trials^3^	Subset	N^4^	Mates	Non-mates	*Z*	*P*	Remarks
Hap2 ph.	≥5%	0.5	1,000	all	28	−43	n/a	n/a	0.015	Reported by Chaix *et al.* [13]
						−43±80	−2±100	−0.41	0.014	Replication
			100,000	all	28	−43±80	−2±100	−0.41	0.022	*P* = 0.022±0.005
				excl.^5^	27	−36±76	−1±99	−0.36	0.052	1 extreme couple excluded
Hap2 un.	≥1%	1	100,000	all	28	−68±120	−3±174	−0.37	0.037	Aggregate *Z* and *P* 6
				Hap2∩3	24	−62±126	−4±173	−0.34	0.073	Couples in Hap2 & Hap3
Hap3 un.	≥1%	1	100,000	Hap2∩3	24	−64±124	−5±171	−0.34	0.067	Couples in Hap2 & Hap3
				Hap3-only^7^	24	−14±132	−1±156	−0.08	0.351	Hap3-only couples, one-sided *P*
				all	45	−36±121	3±164	−0.24	0.143	All Hap3 couples

Excess MHC dissimilarity reported in Phase 2 (Hap2) European mate pairs [13] was not corroborated in an independent Phase 3 (Hap3) sample from the same population. Hap3 included individuals also present in Hap2 (“Hap2∩3”) as well as an independent cohort (“Hap3-only”). Results are ordered as follows: previous findings in Hap2 [13], their replication, and effects of minor modifications (Hap2, phased); for all Hap2 and for Hap2∩3 samples, analyses based on modified methods (Hap2, unphased); analyses of Hap3 genotypes for Hap2∩3 and Hap3-only samples as well as their union. Mean relatedness (±1 standard deviation) is shown for mate pairs and for opposite-sex non-mate pairs, along with effect size (*Z*) and nominal significance (*P*).

1 Ph., phased genotypes; un., unphased genotypes.

2 Het-het score (see Methods).

3 Number of random trials.

4 Couples remaining after exclusions due to the presence of close relatives.

5 Analysis excluding the single most MHC-dissimilar couple.

6 These and subsequent results were based on all possible exclusions due to relatives (see Methods).

7 The *P* value for Hap3-only couples reflects a one-tailed test for the specific hypothesis reported in Hap2 couples.

### Tests of relatedness in Hap3 samples

Unphased genotypes were obtained for Hap2 (release 24) and Hap3 (release 2) populations with parent-child trios. Mate-pair relatedness analyses were only conducted for Europeans and Yorubans due to insufficient numbers of couples in the other Hap 3 population samples (N≤16 following exclusions to avoid the presence of close relatives). Uncalled alleles, which are corrected in phased genotypes [16] but present in unphased genotypes (see Text S1), were skipped in all calculations. Because Hap3 SNP positions are reported for NCBI build 36, we converted the reported MHC coordinates (specified above) using the liftOver program and corresponding chain file (both obtained from the UCSC Genome Bioinformatics web site, http://genome.ucsc.edu) but found them unchanged. MAFs were based on all available samples, including children and unmated individuals, although this was found to have a negligible impact on the number of remaining SNPs (not shown). The minimum MAF was lowered to 1% to conform more closely to the standard definition of common variant, yielding identity coefficients consistent with those based on MAF ≥5% (Supporting Figure 1 in Text S3). Effectively, the minimum number of individuals with minor alleles was 2 for Hap2 and 4 for Hap3. Children and unmated samples were excluded from calculations of mean population-wide identity coefficients (*Q_mean(cohort)_*), and therefore did not influence comparisons of relatedness (*R*) in mate and non-mate pairs. Concordance of results with phased genotypes was verified for Hap2 (Table S2 and Table S3). In Hap3, following the calculation of MAFs, the analysis procedure (detection of relatives, recalculation of *Q_mean_*, etc.) was conducted separately for the subset of Hap3 samples also present in Hap2, for all Hap3 samples, and for samples present in Hap3 only. Because the latter subset of samples allows an independent test of findings reported previously in Hap2, a one-tailed test of significance was conducted in this case for the previously reported hypothesis of interest: 1) relatedness is lower than expected for MHC SNPs in European mates, or 2) relatedness is higher than expected for autosomal SNPs in Yoruban mates.

### Genome-wide relatedness and recombination rate

Chaix *et al.* calculated the recombination rate and mean relatedness between mates *R_mean(mates)_* for 3.6 Mbp segments throughout the genome [13]. To replicate this analysis and apply it to non-mates as well as the Phase 3 population, we obtained recombination rates from the HapMap web site (release 21 for NCBI genome build 35, and release 22 for NCBI build 36), and the centromere coordinates for the respective genome builds from the UCSC Genome Bioinformatics web site. Following Chaix *et al.*, the genome was divided into segments of 3.6 Mbp tiled every 300 Kbp, segments with fewer than 1000 SNPs or overlapping centromeres were excluded, and *R_mean(mates)_* was calculated for each segment based on all SNPs falling within that segment. Additional steps, which may differ from the methods of Chaix *et al.*, were as follows. First, all mated individuals were included in the analyses but pairs of relatives were not included in the mean population-wide identity coefficient, *Q_mean(cohort)_*. Second, the recombination rate for each segment (in cM/Mbp) was calculated from the difference in recombination (cM) between the two data points closest to each end of the segment, with the denominator fixed at 3.6 Mbp. Third, we also conducted this analysis for male-female non-mate pairs; in this case, pairs of relatives were excluded from *Q_mean(cohort)_* and from the mean relatedness for non-mate pairs, *R_mean(non-mates)_*. Lastly, segments overlapping the MHC locus were excluded.

### Correcting for multiple-hypothesis testing

Chaix *et al.* tested four specific locus/population combinations (MHC SNPs in Europeans, MHC SNPs in Yorubans, all autosomal SNPs in Europeans, and all autosomal SNPs in Yorubans), reporting that two of these revealed significant phenomena and two did not; however, no adjustment was made for multiple hypothesis testing [13]. Because there is no clear strategy for modeling dependence between these hypotheses, we adopted the Dunn-Šidák method, which treats hypotheses as mutually independent. The case for treating hypotheses related to the two sets of SNPs as independent of one another is strengthened by scatter plots showing that identity at the MHC locus is poorly correlated with identity overall (Figure 1). Chaix *et al.* reported a *P* value of 0.015 as significant [13], thus we used a nominal significance threshold of *α* = 0.05. For *k* hypotheses tested, the corrected threshold according to the Dunn-Šidák method [17] is *α'* = 1-(1-*α*)^1/*k*^, so that *α'* = 0.0253 for *k* = 2 and *α'* = 0.0127 for *k* = 4.

We note that all *P* values reported in this study are two-sided unless specified otherwise. They are also nominal (uncorrected for multiple testing), so that any correction would further reduce significance.

## Results

### Replication and examination of previous results in Europeans

We successfully replicated each of the specific results of Chaix *et al.* [13] with phased Hap2 genotypes (Table S1). However, we found that MHC dissimilarity of European mate pairs was weak (*Z* = −0.41) and was diminished in significance by small changes in the analysis (Table 1 and Table S2). Significance decreased (from *P* = 0.014 to *P* = 0.022) when the number of random trials was increased (from 1,000 to 100,000), and was lost (*P* = 0.052) upon the subsequent exclusion of the single most MHC-dissimilar couple (Supporting Figure 6 in Text S4). Similarly, the use of median identity and relatedness values instead of means, which should improve robustness to extreme pairs, led to an insignificant result (*P* = 0.288). In addition, results varied depending on which couples were excluded to avoid the presence of related individuals (0.019≤*P*≤0.034). Chaix *et al.* reported results based on an identity measure that assigns a score of 50% to two heterozygous genotypes (het-het  = 50%), but noted that results were similar with het-het  = 100% [13]. Opting for the latter measure in part because it has the intuitive behavior of yielding self-self coefficients of unity (see Text S3), we found results to be consistently weaker, and insignificant (*P* = 0.0546 and *P* = 0.0592) in two of the four possible permutations of excluded couples.

Subsequent analyses were based on unphased genotypes, SNPs with MAF ≥1%, het-het  = 100%, 100,000 random trials, and aggregate values of relatedness and significance calculated for all possible ways of excluding samples such that pairs of related invididuals are eliminated (see Methods). In Hap2 couples, these methods produced results in agreement with those reported previously (Table 1, Table S2 and Table S3). Our conclusions—that reported mate-pair relatedness effects in Europeans and Yorubans are strongly dependent on extreme pairs—were also confirmed using methods that adhered as closely as possible to the original report (not shown but see Figure S1).

### Independent test of excess MHC dissimilarity in European couples

After replicating previously reported results, we sought to test the hypothesis of MHC-dependent mate selection in an independent sample from the same population. Of the 50 couples genotyped in Hap3, 26 “Hap2∩3” couples were also present in Hap2 while 24 “Hap3-only” couples were unique to Hap3 (see Supporting Table 1 in Text S1). This allowed us to assess differences between Hap2 and Hap3 data using Hap2∩3 couples, and also to attempt an independent replication of the reported phenomena using Hap3-only couples.

First, we verified that Hap2 and Hap3 genotypes were concordant for each Hap2∩3 sample (Text S3) and yielded similar results for MHC dissimilarity (Table 1, Table S2, and Text S5), despite the smaller and partially distinct set of SNPs genotyped in Hap3. Second, we determined that the Hap2∩3 and Hap3-only samples were drawn from the same population (Supporting Figure 4 in Text S3). Despite the loss of significance for MHC dissimilarity in the 24 Hap2∩3 couples (*Z* = −0.34, *P*≅0.07), the consistency of results obtained with Hap2 and Hap3 genotypes suggested that Hap3-only couples represented a valid test of previously reported findings.

The 24 independent European mated Hap3-only couples showed negligible and insignificant dissimilarity at the MHC locus relative to random pairs (*Z* = −0.08, one-tailed *P* = 0.351; Figure 1, Table 1). The absence of significance in Hap3 was corroborated with phased genotypes (21 couples; P = 0.497, Z = −0.01) and further confirmed using the original (het-het  = 50%) identity score (not shown). A test of the entire Hap3 cohort (thus including most Hap2 samples) also yielded an insignificant result (*Z* = −0.24, *P* = 0.14; Table 1).

### Sporadic cases of high MHC identity in unmated Europeans

Rare instances of very high MHC identity were observed among unrelated non-mate pairs in Hap2 Europeans but not among couples (Figure 1 and Figure 2), suggesting the possibility of a bias against extremely high MHC similarity in mate pairs (or their offspring; see Discussion), rather than a preference toward dissimilarity. In both the Hap2 and Hap3-only subsets, we observed a possible depletion of high MHC similarity in mate pairs relative to non-mate pairs (Figure 2). This potential excess of high MHC similarity in European non-mate pairs is not seen in other populations (Figure S2) or in autosomal similarity (not shown). However, sample sizes are too small to permit a rigorous test of this hypothesis.

### Independent test of excess autosomal relatedness in Yoruban couples

We also re-examined the previous report of excess autosomal relatedness among mated pairs in the Yoruban population [13]. We first replicated previous results in Hap2 couples, and obtained equivalent results using modified methods described above (Table S1 and Table 2). In addition, based on Hap2 genotypes, the effect remained significant for the 24 Hap2 couples that were also genotyped in Hap3 (“Hap2∩3”; *P* = 0.01, *Z* = 0.56; Table 2). However, an examination of these same couples using Hap3 genotypes did not confirm the previous finding (*P* = 0.13, *Z* = 0.33), even when only common SNPs were considered (Table 2 and Table S3; see Text S6 for a discussion of this discrepancy). Finally, we examined the 26 independent couples present in Hap3 but not Hap2 (“Hap3-only”) and found them to confirm the previous finding of excess similarity among mate pairs (Table 2).

**Table 2:** Summary of results for autosomal relatedness in HapMap Yorubans.

	Methods			Mate pairs		Relatedness x10^5^		Significance		
Genotypes	MAF	Het	Trials	Subset	N	Mates	Non-mates	*Z*	*P*	Remarks
Hap2 ph.	≥5%	0.5	1,000	all	27	185	n/a	n/a	<10^−3^	Reported by Chaix *et al.* [13]
			100,000	all	27	185±257	3±229	0.80	6×10^−5^	Replication, 10^5^ random trials
Hap2 un.	≥1%	1	100,000	all	27	205±366	3±361	0.56	0.004	Aggregate *Z* and *P*
				Hap2∩3	24	186±391	−12±355	0.56	0.011	Couples in Hap2 & Hap3
Hap3 un.	≥1%	1	100,000	Hap2∩3	24	109±442	−4±346	0.33	0.126	Couples in Hap2 & Hap3
				Hap3-only	28	165±526	−5±407	0.42	0.016	Hap3-only couples, one-sided *P*
				all	51	146±472	−4±372	0.41	0.005	All Hap3 couples

**Figure 3:**
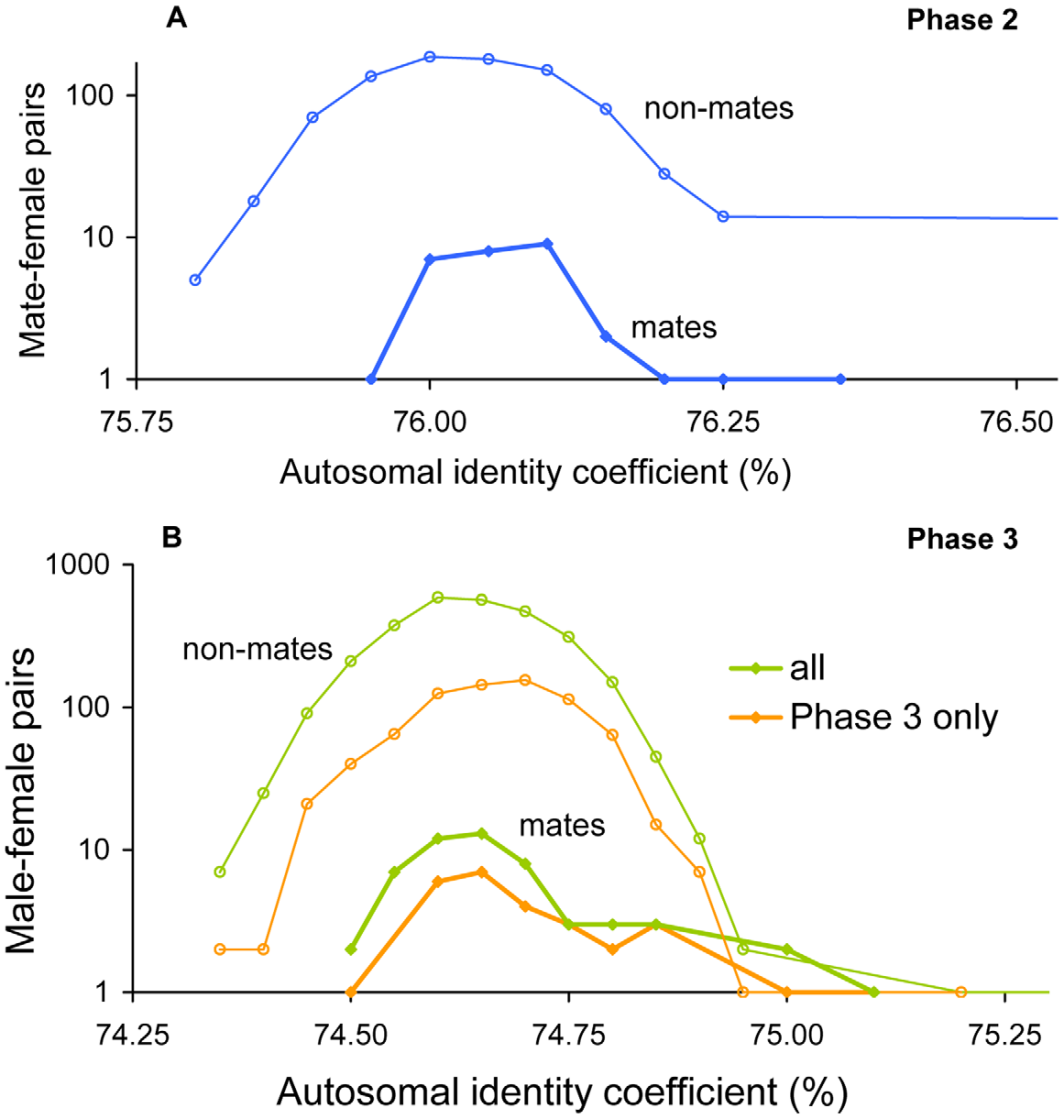
Overall identity in HapMap Yorubans. Distributions of identity coefficients based on all autosomal SNPs are shown for mates and for non-mate pairs in (A) Phase 2 (30 couples), and (B) Phase 3 (“all”; 54 couples), as well as the subset of Phase 3 couples not genotyped in Phase 2 (“Phase 3 only”; 28 couples). Coefficients were lower in Phase 3 because fewer SNPs with low minor allele frequencies were genotyped.

Excess autosomal similarity reported in Phase 2 (Hap2) Yoruban mate pairs [13] was confirmed in an independent Phase 3 (Hap3) sample from the same population. However, Hap2 and Hap3 genotypes yielded discrepant results for Hap2∩3 couples (even if the same SNPs were examined; see Table S3). The difference in relatedness between mates and non-mate pairs appears to be driven by a subset of the sample (see Figure 3). The format follows that of Table 1.

We observed that both the Hap2 and Hap3-only samples contained a small number of mate pairs with unusually high similarity (Figure 3), suggesting that they may be relatives. The distribution of mate-pair identity coefficients for all Hap3 samples has a shoulder that suggests an underlying mixture of two types of couples (Figure 3). Thus, an enrichment for autosomal similarity previously reported in Yoruban mate pairs [13] is confirmed, but may be driven by a subset of the couples.

### Genome-wide relatedness and recombination rate

In Hap2 Europeans, Chaix *et al.* found that mean relatedness between mates at the MHC locus was lower than or equal to relatedness between mates at 99.6% of similarly-sized genomic segments, or 99.9% of segments with the same or lower recombination rate as the MHC locus [13]. Thus, the conclusion that relatedness at the MHC locus was extreme relative to other genomic loci is well supported. However, this observation could be explained by phenomena other than mate selection, e.g., by elevated positive selection leading to heightened diversity at the MHC locus. This explanation is supported by the high degree of polymorphism observed at the MHC locus [1]. An observation of relatedness between non-mates at the MHC locus that is also systematically lower than other genomic loci would argue against a mate selection explanation. We therefore performed a similar analysis for non-mate pairs, which was not presented in the previous report [13].

We first replicated the reported analysis as closely as possible (see Methods and Text S7), then applied it to opposite-sex non-mate pairs in Hap2 and to mates and non-mates in Hap3. In Hap2 Europeans, although we obtained slightly higher numbers of segments than reported with lower relatedness than the MHC locus, we found that the MHC locus is only slightly less extreme in a genome-wide analysis in non-mates than in mates: mean relatedness for non-mates at the MHC locus was lower than or equal to relatedness at 96.9% of all segments (compared with 99.0% for mate pairs) and 94.3% of segments with equal or lower recombination rate (as opposed to 97.1% for mate pairs; Supporting Table 4 in Text S7). In the combined Hap3 European population, which showed a slight but insignificant difference in mean MHC relatedness between mates and random couples (*Z* = −0.24, *P* = 0.143; Table 1), only 1.6% of segments in mates but 91.0% in non-mates had lower mean relatedness than the MHC locus. Rather than suggesting that the MHC locus is unique, these results appear to simply reflect the large standard deviations observed for MHC relatedness in both mates and non-mates (Table 1). In Hap3 Yorubans, the MHC locus is extreme in opposite directions in mates and non-mates: mean relatedness is lower at 94.6% of all loci than at the MHC locus in mates, but lower in only 1.6% of loci in non-mates. Given that there is no evidence in Yorubans of a significant difference in MHC relatedness between mates and non-mates, these results may be explained by the previous finding [13] (confirmed here) that Yorubans exhibit a broad mate-dependent shift in relatedness across autosomal loci.

### Adjustment for multiple hypothesis testing

Standards vary on when, whether and how to correct significance tests when multiple hypotheses are examined. Chaix *et al.* tested four hypotheses regarding the difference between observed and expected relatedness in mate pairs, namely regarding autosomal and MHC relatedness in Yorubans and in Europeans, but did not report any corrections for multiple hypotheses [13]. We examined the effect of correcting previously reported nominal *P* values for multiple hypothesis testing. First, given that results were presented separately for each population [13] and that each finding was of interest on its own, one might reasonably consider that each population represents an independent hypothesis test (of excess MHC dissimilarity in mates relative to random couples) and therefore warrants correction. For two hypotheses, the corrected significance threshold is *α'* = 0.0253 (see Methods), so that the previously-reported MHC relatedness in Hap2 European mates retains significance (*P* = 0.015≤*α'*).

Analyses of autosomal SNPs were introduced in the previous report as negative controls; however, the excess autosomal relatedness in Yoruban mates was presented as a significant result *per se* [13]. Therefore, another correction may have been warranted for the two sets of SNPs assayed (MHC and autosomal). For four hypotheses, the corrected significance threshold is *α'* = 0.0127, so that MHC relatedness in Hap2 European mates would no longer differ significantly from expectation (*P* = 0.015≥*α'*), whereas overall relatedness in Yoruban couples would (*P*<0.001).

## Discussion

We found that the previously reported MHC dissimilarity among Hap2 European-American mate pairs [13] is apparent but not robustly supported by the underlying genotypic evidence. In addition, the effect essentially disappears in Hap3 for a similar number of independent couples from the same population, and is weak and insignificant for the combined Hap3 cohort. We cannot explain the discrepancy based on differences in SNPs assayed or by the imputation of missing alleles in phased data, given that Hap2 and Hap3 genotypes yield concordant results for the same couples (Hap2∩3), as do phased and unphased genotypes. In addition, Hap2 and Hap3 samples appear to be drawn from the same population (Supporting Figure 4 in Text S3), suggesting an explanation other than population structure. The fact that the MHC dissimilarity in Hap2 couples becomes marginal upon minor modifications in the methods and included samples suggests that the result was weaker than reported and did not represent a significant difference between mate pairs and non-mate pairs.

This conclusion is supported by the observation that a stringent correction for multiple-hypothesis testing renders the original finding of MHC dissimilarity insignificant, even given the previously-reported nominal (uncorrected) *P* value. It is certainly true that multiple testing corrections are a matter of ongoing debate and diverse preference, and overly conservative approaches can lead to a substantial loss of power. However, even stricter approaches to multiple testing can be argued. For example, the authors might also have corrected for the several instances wherein they tested two alternative methods and chose one.

Although our analysis of HapMap genotypes does not support a broad and significant dependence of mate selection on MHC, a weak effect is apparent in Hap2 even when the most extreme couples are excluded (Supporting Figure 6 in Text S4). In addition, the apparent depletion of very high MHC identity coefficients among mates (Figure S2) hints that mate selection may disfavor extreme MHC similarity. Unfortunately, too few samples are currently available to pursue this hypothesis. Following the previous report [13], we considered the entire MHC locus as a single unit. Because this genomic region contains many genes and exhibits variable rates of recombination [1], it is possible that an examination of MHC genotypes at a finer scale would reveal a correlation with mate selection. Given the need to adjust for multiple hypothesis testing, and the likelihood (based on HapMap samples) that any effects would be subtle, a rigorous investigation of this question will require many more samples.

If mates were found to differ from non-mates in MHC relatedness, in these or other populations, we note that this phenomenon need not stem from mate selection alone, particularly if only couples with children are considered. If offspring with certain MHC allele combinations survive preferentially, exclusion of mated couples without children could yield a non-random MHC similarity distribution amongst the remaining couples. This idea is supported by the increase in MHC heterozygosity of mouse embryos following viral infection of the parents [18].

The reported preference of Hap2 Yoruban individuals for mates more similar to themselves overall [13] was not robust to changing the source of genotype data. However, it was corroborated in an independent set of Hap3 couples. As with MHC dissimilarity in Europeans, the mate-dependent autosomal similarity effect detected in Yorubans appears to be driven by a small subset of pairs. Larger studies will be required to shed further light on these hypotheses.

## Supporting Information

Figure S1 Identity coefficients of HapMap couples using original methods and phased genotypes.
(0.20 MB PDF)

Figure S2 Sporadic cases of high MHC similarity in HapMap European non-mate pairs.
(0.10 MB PDF)

Table S1 Replication of previous findings in Hap2 mate pairs.
(0.06 MB PDF)

Table S2 Detailed results for MHC relatedness in European couples.
(0.08 MB PDF)

Table S3 Detailed results for autosomal relatedness in Yoruban couples.
(0.08 MB PDF)

Text S1 Overview of procedure and datasets.
(0.10 MB PDF)

Text S2 Sample pairs identified as relatives.
(0.08 MB PDF)

Text S3 Modification of methods, tests of concordance, and application to Hap3 cohorts.
(0.55 MB PDF)

Text S4 Critical impact of an extreme couple on mate-pair MHC dissimilarity.
(0.10 MB PDF)

Text S5 Analysis of Hap2∩3 couples with Hap2 and Hap3 genotypes.
(0.12 MB PDF)

Text S6 Discrepancy detected in Hap2 Yorubans.
(0.09 MB PDF)

Text S7 Genome-wide relatedness and recombination rate.
(0.09 MB PDF)
