# Supplementary material for: Absence of Evidence for MHC–Dependent Mate Selection within HapMap Populations
Source: PLoS Genet. 2010 Apr 29;6(4):e1000925. doi: 10.1371/journal.pgen.1000925 (PMC2861700; doi:10.1371/journal.pgen.1000925)
Supplement: Figure S1 — Identity coefficients of HapMap couples using original methods and phased genotypes. (0.20 MB PDF) [file pgen.1000925.s001.pdf]

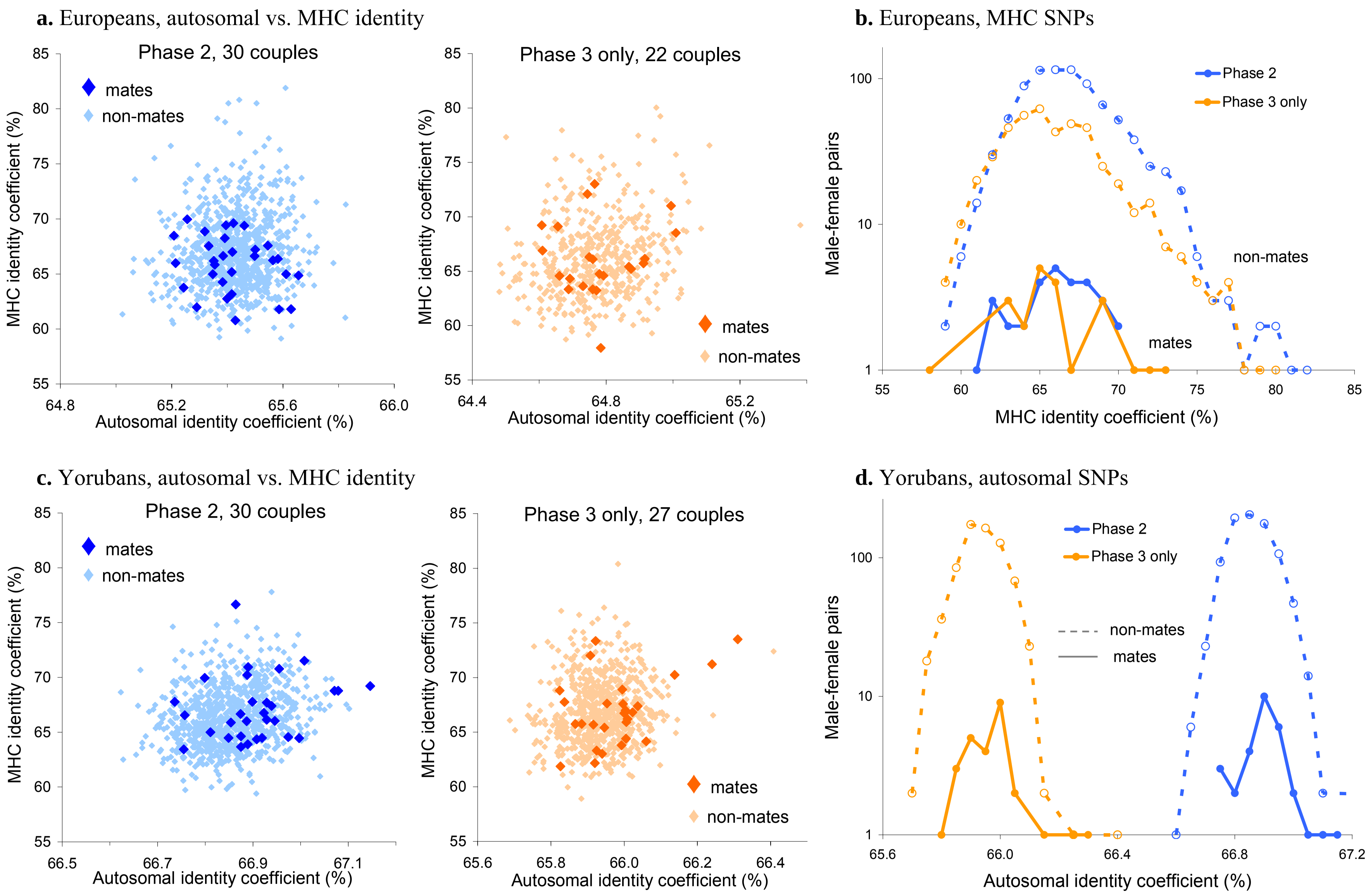

Figure S1. Identity coefficients of HapMap couples using original methods and phased genotypes. Plots are based on phased genotypes, SNPs with MAF≥5%, and het-het=50%. Results are similar to those obtained with modified methods, and reveal only minor differences between mates and non-mates. Refer Supporting Table 1 in Text S1 for differences in sample sizes of Phase 3 phased and unphased data. Autosomal identity coefficients were lower in Phase 3 because fewer SNPs with low minor allele frequencies were genotyped.
