# Supplementary material for: Absence of Evidence for MHC–Dependent Mate Selection within HapMap Populations
Source: PLoS Genet. 2010 Apr 29;6(4):e1000925. doi: 10.1371/journal.pgen.1000925 (PMC2861700; doi:10.1371/journal.pgen.1000925)
Supplement: Figure S2 — Sporadic cases of high MHC similarity in HapMap European non-mate pairs. (0.10 MB PDF) [file pgen.1000925.s002.pdf]

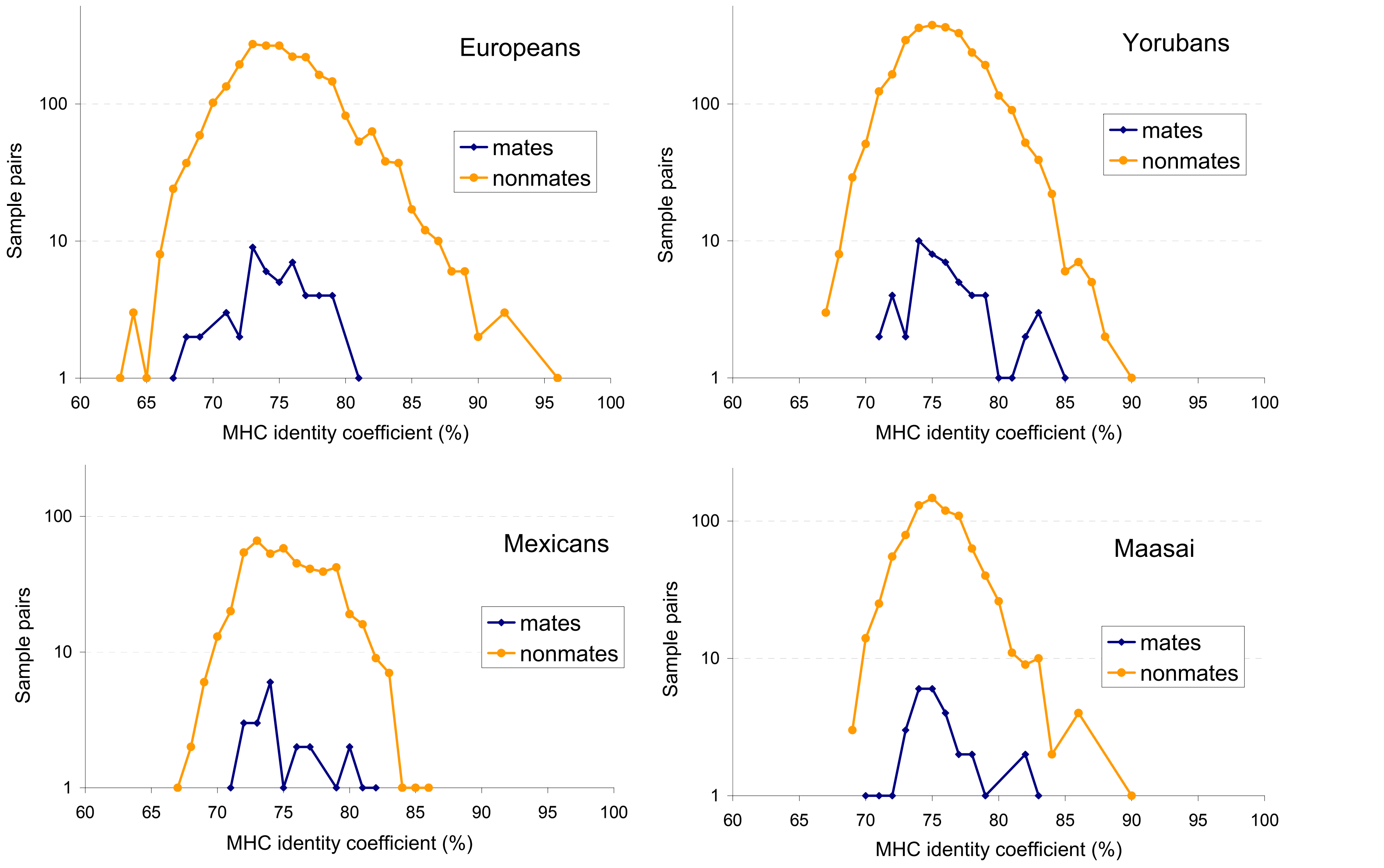

Figure S2. Sporadic cases of high MHC similarity in HapMap European non-mate pairs. For four of the five HapMap Phase 3 populations with parent-child trios, distributions of identity coefficients are shown for mate pairs and male-female non-mate pairs (excluding close relatives); African-Americans were omitted due to the small sample size. The occurrence of high identity coefficients among non-mate pairs is most apparent in Europeans.
