# Supplementary material for: Absence of Evidence for MHC–Dependent Mate Selection within HapMap Populations
Source: PLoS Genet. 2010 Apr 29;6(4):e1000925. doi: 10.1371/journal.pgen.1000925 (PMC2861700; doi:10.1371/journal.pgen.1000925)
Supplement: Table S1 — Replication of previous findings in Hap2 mate pairs. (0.06 MB PDF) [file pgen.1000925.s003.pdf]

Population	N[a]	SNPs	Source	Mean relatedness	*P*
Europeans	28	All[b]	Reported[c]	-0.00016	0.739
			Replication	-0.00016	0.806
		MHC	Reported	-0.043	0.015
			Replication	-0.043	0.014
Yorubans	27	All	Reported	0.00185	<0.001
			Replication	0.00185	<0.001
		MHC	Reported	0.015	0.23[d]
			Replication	0.015	0.308

Table S1. Replication of previous findings in Hap2 mate pairs. Mean relatedness for mate pairs is shown for each population and each set of SNPs under consideration, along with the nominal significance of its deviation from expectation (*P*). Highlights indicate results previously reported to be significant [13], which are the focus of this study. In the interest of completeness, corresponding figures are also shown for results not examined further. [a]Of the 30 couples in each population, the number remaining after exclusions due to the presence of relatives. [b]All autosomal SNPs. [c]Reported by Chaix *et al.* [13]. [d]As reported.
