# Supplementary material for: Absence of Evidence for MHC–Dependent Mate Selection within HapMap Populations
Source: PLoS Genet. 2010 Apr 29;6(4):e1000925. doi: 10.1371/journal.pgen.1000925 (PMC2861700; doi:10.1371/journal.pgen.1000925)
Supplement: Table S2 — Detailed results for MHC relatedness in European couples. (0.08 MB PDF) [file pgen.1000925.s004.pdf]

Genotypes	Couples[a]		SNPs		Het-het[b]	Relatedness				Z	P	Remarks
						Mates		Non-mates				
	Subset	N	Subset	MAF		mean	s.d.	mean	s.d.			
Hap2, Phased	all	28	all	≥5%	.5	-0.043	n/a	n/a	n/a	n/a	0.015	Reported[c]
						-0.043	0.080	-0.0020	0.100	-0.41	0.014	Replication
						-0.020	0.061	-0.0021	0.058	-0.31	0.288	Median[d]
						-0.043	0.080	-0.0020	0.100	-0.41	0.0218	100,000 trials[e]
						-0.043	0.081	-0.0020	0.100	-0.40	0.0217	All in MAF[f]
						-0.043	0.078	-0.0019	0.098	-0.42	0.0190	Permutations of excluded couples[g]
						**-0.043**	**0.081**	**-0.0020**	**0.100**	**-0.40**	**0.0226**	
						-0.040	0.076	-0.0026	0.099	-0.38	0.0304	
						-0.040	0.080	-0.0027	0.101	-0.37	0.0335	
					1	**-0.068**	**0.121**	**-0.0029**	**0.173**	**-0.38**	**0.0348**	Permutations of excluded couples[g]
						-0.068	0.123	-0.0026	0.174	-0.37	0.0371	
						-0.062	0.120	-0.0037	0.171	-0.34	0.0546	
						-0.061	0.122	-0.0036	0.172	-0.33	0.0592	
				≥1%	1	-0.062	0.123	-0.0033	0.172	-0.34	0.0530	Aggregate mean, s.d., P, Z[h]
Hap2, unphased	all	28	all	≥1%	1	-0.068	0.120	-0.0032	0.174	-0.37	0.0368	
	2∩3	24				-0.063	0.126	-0.0039	0.173	-0.34	0.0727	
			2∩3			-0.060	0.129	-0.0047	0.170	-0.33	0.0810	
Hap3, unphased	2∩3	24	all			-0.064	0.124	-0.0052	0.171	-0.34	0.0670	
			2∩3			-0.063	0.127	-0.0049	0.170	-0.34	0.0670	
	3-only	24	all			-0.014	0.132	-0.0011	0.156	-0.08	0.3512	One-tailed P
			2∩3			-0.020	0.133	-0.0008	0.157	-0.12	0.2792	

Table S2. Detailed results for MHC relatedness in European couples. The mean and standard deviation (s.d.) of relatedness for mate and non-mate pairs are shown, along with the nominal significance (P) and effect size (Z). [a]Subset of couples included for corresponding HapMap Phase, and similarly for SNPs. [b]Identity score assigned to two heterozygous genotypes. [c]Results previously reported by Chaix *et al.* [13]. [d]Median values were used instead of means throughout methods; in this case, values in the mean and s.d. columns are in fact median and median absolute difference, respectively. [e]For these and all subsequent results, 100,000 random trials were conducted. [f]For these and all subsequent results, all samples were included in the calculation of MAF. [g]Results are sorted in decreasing order of significance, and bold type indicates results obtained for the combination used in the previous report [13]. [h]Results are aggregates derived from all permutations of excluded couples.
