# Supplementary material for: Absence of Evidence for MHC–Dependent Mate Selection within HapMap Populations
Source: PLoS Genet. 2010 Apr 29;6(4):e1000925. doi: 10.1371/journal.pgen.1000925 (PMC2861700; doi:10.1371/journal.pgen.1000925)
Supplement: Table S3 — Detailed results for autosomal relatedness in Yoruban couples. (0.08 MB PDF) [file pgen.1000925.s005.pdf]

| Genotypes | Couples | | SNPs | | Het-het | Relatedness | | | | $Z$ | $P$ | Remarks |
|---|---|---|---|---|---|---|---|---|---|---|---|---|
| | | | | | | Mates | | Non-mates | | | | |
| | Subset | N | Subset | MAF | | mean | s.d. | mean | s.d. | | | |
| Hap2, Phased | all | 27 | all | ≥5% | .5 | 0.00185 | n/a | n/a | n/a | n/a | <0.001 | Reported |
| | | | | | | 0.00185 | 0.003 | 0.00003 | 0.002 | 0.80 | <0.001 | Replication |
| | | | | | | 0.00150 | 0.001 | 0.00004 | 0.002 | 0.90 | 0.007 | Median |
| | | | | | | 0.00185 | 0.003 | 0.00003 | 0.002 | 0.80 | 0.00006 | 100,000 trials |
| | | | | | | 0.00185 | 0.003 | 0.00003 | 0.002 | 0.80 | 0.00010 | All in MAF |
| | | | | | | **0.00185** | **0.003** | **0.00003** | **0.002** | **0.80** | **0.00002** | Permutations of excluded couples |
| | | | | | | 0.00175 | 0.003 | -0.00001 | 0.002 | 0.77 | 0.00011 | |
| | | | | | | 0.00179 | 0.003 | 0.00001 | 0.002 | 0.76 | 0.00011 | |
| | | | | | | 0.00168 | 0.003 | -0.00002 | 0.002 | 0.73 | 0.00021 | |
| | | | | | | 0.00167 | 0.003 | 0.00004 | 0.002 | 0.71 | 0.00026 | |
| | | | | | | 0.00157 | 0.003 | 0.00001 | 0.002 | 0.68 | 0.00044 | |
| | | | | | | 0.00161 | 0.003 | 0.00002 | 0.002 | 0.67 | 0.00062 | |
| | | | | | | 0.00150 | 0.003 | 0.00000 | 0.002 | 0.64 | 0.00096 | |
| | | | | | 1 | **0.00270** | **0.004** | **0.00004** | **0.003** | **0.77** | **0.00006** | Permutations of excluded couples |
| | | | | | | 0.00267 | 0.004 | 0.00003 | 0.004 | 0.75 | 0.00009 | |
| | | | | | | 0.00257 | 0.004 | -0.00001 | 0.003 | 0.74 | 0.00017 | |
| | | | | | | 0.00253 | 0.004 | -0.00001 | 0.004 | 0.71 | 0.00024 | |
| | | | | | | 0.00245 | 0.004 | 0.00006 | 0.003 | 0.69 | 0.00028 | |
| | | | | | | 0.00243 | 0.004 | 0.00004 | 0.004 | 0.67 | 0.00043 | |
| | | | | | | 0.00233 | 0.004 | 0.00003 | 0.004 | 0.66 | 0.00064 | |
| | | | | | | 0.00230 | 0.004 | 0.00002 | 0.004 | 0.63 | 0.00113 | |
| | | | | ≥1% | 1 | 0.00248 | 0.004 | 0.00002 | 0.003 | 0.71 | 0.00035 | Aggregate mean, s.d., $P$, $Z$ |
| Hap2, unphased | all | 27 | all | ≥1% | 1 | 0.00205 | 0.004 | 0.00003 | 0.004 | 0.56 | 0.00356 | |
| | 2∩3 | 24 | | | | 0.00186 | 0.004 | -0.00012 | 0.004 | 0.56 | 0.01088 | |
| | | | 2∩3 | | | 0.00145 | 0.004 | -0.00003 | 0.003 | 0.44 | 0.03377 | |
| Hap3, unphased | 2∩3 | 24 | all | | | 0.00109 | 0.004 | -0.00004 | 0.003 | 0.33 | 0.12554 | |
| | | | 2∩3 | | | 0.00111 | 0.004 | -0.00003 | 0.003 | 0.35 | 0.09169 | |
| | 3-only | 28 | all | | | 0.00165 | 0.005 | -0.00005 | 0.004 | 0.42 | 0.01648 | One-tailed $P$ |
| | | | 2∩3 | | | 0.00178 | 0.005 | -0.00005 | 0.004 | 0.49 | 0.00659 | |

**Table S3. Detailed results for autosomal relatedness in Yoruban couples.** The format follows that of Table S2.
