# Supplementary material for: Absence of Evidence for MHC–Dependent Mate Selection within HapMap Populations
Source: PLoS Genet. 2010 Apr 29;6(4):e1000925. doi: 10.1371/journal.pgen.1000925 (PMC2861700; doi:10.1371/journal.pgen.1000925)
Supplement: Text S1 — Overview of procedure and datasets. (0.10 MB PDF) [file pgen.1000925.s006.pdf]

## Text S1. Overview of procedure and data sets

Details in Supporting Information are presented in the following sequence:

1) Replication of the findings of Chaix *et al.* using Hap2 phased data.
2) Verification of concordance of results using Hap2 unphased data and modified methods.
3) Analyses applied to samples common to Hap2 and Hap3, using all SNPs in each phase then only common SNPs.
4) Analyses conducted for couples only present in Hap3.

**HapMap phases and phased genotypes.** The use of the term "phase" in two different contexts in the HapMap data necessitates a brief explanation. First, the genotypes analyzed in this study pertain to two broad stages of the HapMap project, Phase 2 and Phase 3; we refer to these as Hap2 and Hap3, respectively. Briefly, fewer SNPs were assayed in more samples in Hap3, including the majority of Hap2 individuals. Second, HapMap genotypes are periodically "phased", meaning that haplotypes are inferred and each allele in an individual is categorized as either transmitted or untransmitted. In unphased genotypes, the two alleles are reported *per se*, without information regarding haplotypes. Thus, phased and unphased genotypes are available for Hap2 and Hap3. One difference between these types of data is that uncalled alleles (N) present in unphased genotypes are typically corrected in phased data [16].

The analyses of Chaix *et al.* on Hap2 couples [13] were based on phased genotypes (release 21), although phase information was not used (R. Chaix, personal communication). We opted to analyze unphased genotypes instead because, at the time of our study, a more recent Hap2 release was available, and more Hap3 samples were available, both in total and in common with Hap2 (Supporting Table 1).

| HapMap Phase | Genotypes | | Yorubans | | | Europeans | | |
|---|---|---|---|---|---|---|---|---|
| | Type | Release | 2∩3 | 3-only | Total | 2∩3 | 3-only | Total |
| Hap2 | Unphased | 24 | | | 30 | | | 30 |
| | Phased | 21 | | | 30 | | | 30 |
| Hap3 | Unphased | 2 | 26 | 28 | 54 | 26 | 24 | 50 |
| | Phased | 2 | 23 | 27 | 50 | 22 | 22 | 44 |

**Supporting Table 1. Couples in HapMap genotype data.** The numbers of couples present in each phase, common to both phases (2∩3), and unique to Hap3 (3-only), if applicable, are given for unphased and phased genotypes. In Hap2, the same couples were present in phased and unphased genotypes. In Hap3, phased genotypes contained a subset of samples with unphased genotypes.

**Inclusion of phased data.** Although we concentrated on unphased genotypes because more samples were available in Hap3, we also examined phased data, for two reasons: first, in order to replicate the results of Chaix *et al.* [13] exactly; and second, to minimize the possibility that differences between phased and unphased data might influence the applicability of methods and results to Hap3 individuals. In Yorubans, we uncovered a discordance between Hap2 and Hap3, using the same individuals and SNPs, whether we used phased or unphased genotypes (Text S6).
