# Supplementary material for: Absence of Evidence for MHC–Dependent Mate Selection within HapMap Populations
Source: PLoS Genet. 2010 Apr 29;6(4):e1000925. doi: 10.1371/journal.pgen.1000925 (PMC2861700; doi:10.1371/journal.pgen.1000925)
Supplement: Text S2 — Sample pairs identified as relatives. (0.08 MB PDF) [file pgen.1000925.s007.pdf]

Text S2. Sample pairs identified as relatives

In the previous report by Chaix *et al.* [13], the three European and three Yoruban sample pairs considered close relatives were those identified by HapMap scientists [14] as having a relatedness coefficient $f \geq \sim 1/32$. The inclusion of Hap3 samples in our analysis necessitated *de novo* detection of relatives; we based this step on the relatedness coefficient R (see Methods) but chose R thresholds that maximized concordance with f. We first calculated R for all pairs of samples using the het-het = 50% score and SNPs with MAF ≥ 1% (see Methods). For five of the six sample pairs mentioned above, this yielded values similar to f ($R \geq 1/33$; Supporting Table 2). The remaining pair (NA07022-NA06993) was slightly below the threshold ($R = 1/40$), but was effectively captured in that NA07022 and NA07056 are mates, and both are related to NA06993.

Our analyses were based on the het-het = 100% score (see Methods), and we therefore also used het-het = 100% to calculate R for all sample pairs in Hap2 and Hap3, again using SNPs with MAF ≥ 1%. Based on these results (Supporting Table 2), we used the threshold R ≥ 1/22 to detect pairs of relatives in Hap3.

Supporting Table 2 lists the pairs of samples identified as relatives:

Population	Samples		f_{HapMap} [b]	Hap2		Hap3, $R_{het=1}$	
	A [a]	B		$R_{het=0.5}$	$R_{het=1}$	Unphased	Phased
Europeans	NA07022	NA06993	~1/32	1/40	1/27	1/27	
	NA07056	NA06993	~1/32	1/33	1/21	1/21	
	NA12264	NA12155	~1/32	1/33	1/21	1/21	1/21
	NA12043	NA07031				1/18	
	NA12760	NA12830				1/22	
	NA12813	NA07045				1/2.4	1/2.4
Yorubans	NA19101	NA19092	~1/16	1/19	1/12		
	NA18913	NA19238	~1/4	1/4	1/2.6	1/2.6	
	NA19130	NA19192	~1/8	1/8	1/5	1/5	1/5
	NA19200	NA19178				1/8	1/8

Supporting Table 2. Sample pairs identified as relatives. For each pair of relatives, relatedness values are indicated for the sets of genotypes in which both samples appear. All other pairs of samples were less related than those shown. [a]For Hap2 pairs of relatives, the sample chosen for exclusion by Chaix et al. [13] (R. Chaix, personal communication) is listed first; these samples and their mates were excluded in the replication of previously reported results. [b]Values reported in [14].
