# Supplementary material for: Absence of Evidence for MHC–Dependent Mate Selection within HapMap Populations
Source: PLoS Genet. 2010 Apr 29;6(4):e1000925. doi: 10.1371/journal.pgen.1000925 (PMC2861700; doi:10.1371/journal.pgen.1000925)
Supplement: Text S3 — Modification of methods, tests of concordance, and application to Hap3 cohorts. (0.55 MB PDF) [file pgen.1000925.s008.pdf]

Text S3. Modification of methods, tests of concordance, and application to Hap3 cohorts

This section describes: 1) steps taken to ensure that changes in methods would not distort results; 2) tests of concordance of Hap2 vs. Hap3 genotypes (i.e., for samples present in both studies, or Hap2∩3), and of phased vs. unphased genotypes; and 3) comparisons of Hap2∩3 samples with Hap3-only samples. The primary intent of these comparisons was to ascertain the validity of testing the findings of mate-pair (dis)similarity in independent Hap3 cohorts from the same respective populations.

In Hap2 individuals, identity coefficients were concordant with those obtained with the original methods if the MAF threshold is lowered from 5% to 1% (Supporting Figure 1) or if unphased genotypes were used in place of phased genotypes (Supporting Figure 2 and Figure S1).

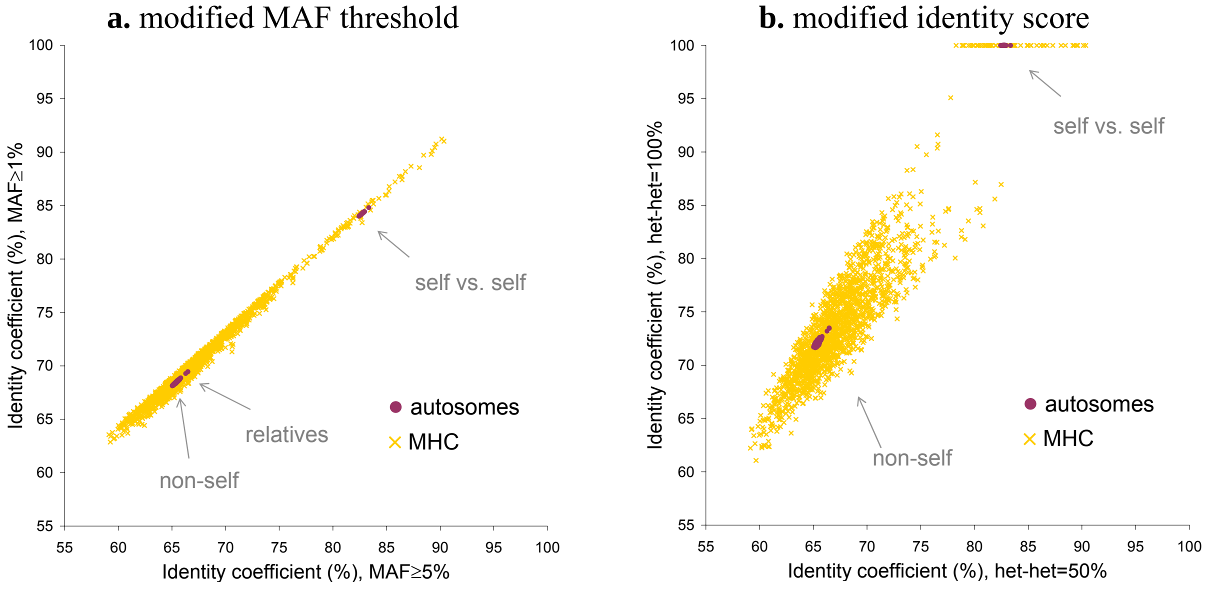

Supporting Figure 1. Modification of methods. Identity coefficients are concordant with MAF thresholds of 5% and 1% (a), but discordant with het-het scores of 50% and 100% (b). Autosomal and MHC identity coefficients calculated with original methods and data (phased genotypes, MAF≥5%, het-het=50%) are plotted against those calculated with the MAF threshold lowered to 1% (a) or a het-het score of 100% (b), for all pairs among Hap2 European samples. Results were similar for Yorubans (not shown).

Chaix *et al.* reported results obtained with het-het=50%, but noted that results were concordant with het-het=100% [13]. Based on this equivalence, we opted to use het-het=100%, which we found more intuitive and better suited for determining concordance between different versions of genotypes. For example, the theoretical maximum identity coefficient is unity when het-het=100%, allowing the concordance of Hap2 and Hap3 genotypes for the same sample to be readily assessed (Supporting Figure 3). In contast, self-self identity coefficients are significantly lower than 1 with het-het=50%, and particularly variable for MHC SNPs (Supporting Figure 1).

With the MAF threshold lowered to 1% and het-het=100%, we calculated identity coefficients for non-self Hap2∩3 sample pairs in four ways, namely with phased and unphased genotypes from Hap2 and Hap3. We found broad concordance among these data sets (Supporting Figure 2):

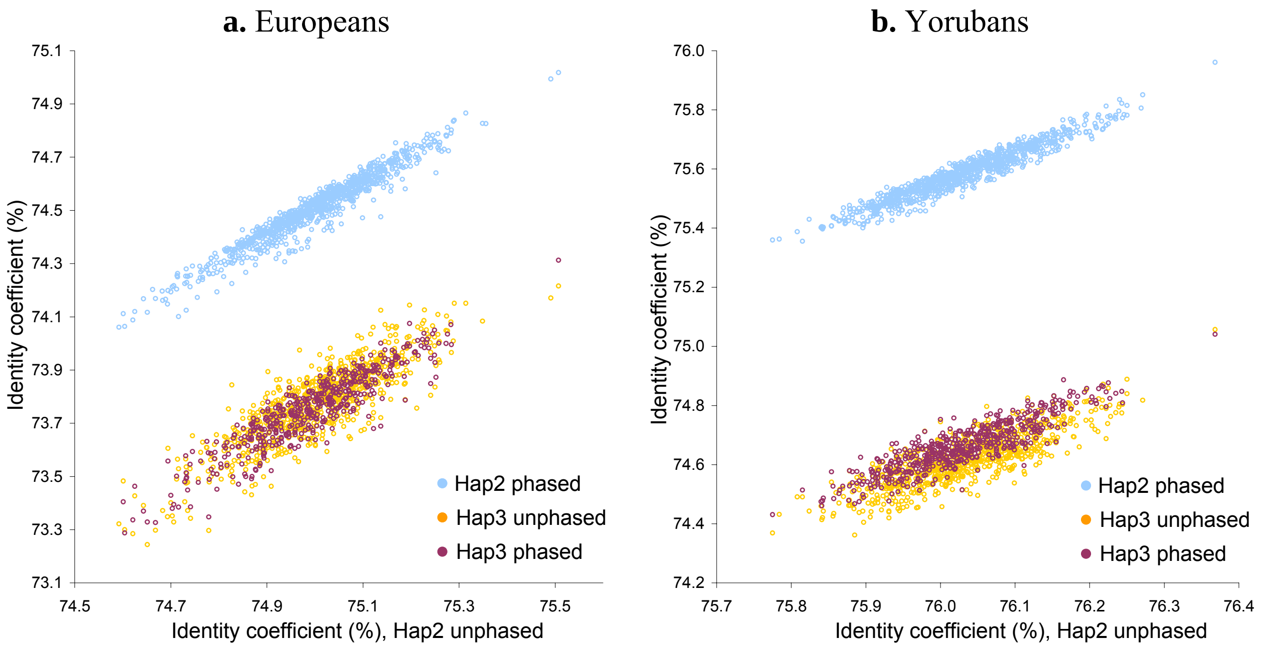

Supporting Figure 2. Concordance of autosomal identity coefficients with modified methods. Coefficients calculated from phased and unphased genotypes were similar, as were coefficients calculated with Hap2 and Hap3 genotypes. Non-self coefficients are shown (MAF≥1% and het-het=100%) for all Hap2∩3 sample pairs. Coefficients were lower in Phase 3 because fewer SNPs with low minor allele frequencies were genotyped.

Based on these results, and because unphased genotypes were available for a greater number of samples in Hap3 and were available in a more recent release than phased genotypes in Hap2, we conducted our analyses using unphased genotypes.

Next, we conducted several tests to assess the validity of extending the analyses to Hap3 populations. First, to probe for any biases arising from differences in HapMap methods (e.g. SNPs assayed, processing of samples and genotypes), we calculated the self-self identity coefficient of each Hap2∩3 sample using Hap2 and Hap3 genotypes (i.e., based on SNPs assayed in both phases). We found high concordance of Hap2 and Hap3 genotypes for the same sample (Supporting Figure 3), with some variability for MHC coefficients:

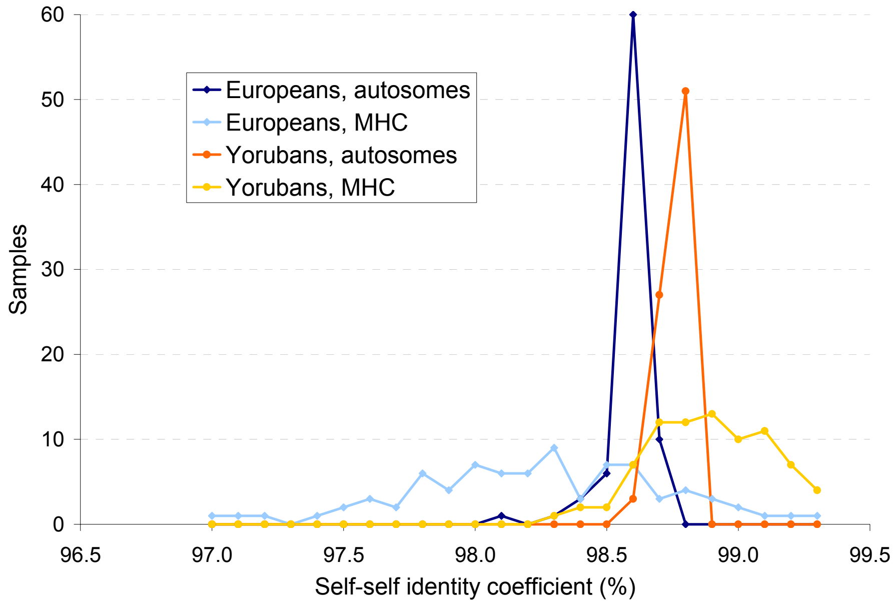

Supporting Figure 3. Correspondence of Hap2 and Hap3 genotypes. For every sample present in Hap2 and Hap3, the Hap2-Hap3 self-self identity coefficient was calculated using unphased genotypes, common SNPs with MAF≥1% in both data sets, a het-het score of 100%, and either all autosomal SNPs or only those within the MHC locus. For each population, N=81, including 26 mate pairs as well as children and unmated samples. Autosomal self-self identity coefficients from Hap2 to Hap3 are close to unity (the theoretical maximum using the het-het=1 score), while self-self coefficients based on MHC SNPs alone exhibit greater variability.

Second, because most Hap2 samples were assayed again in Hap3, we compared the Hap2∩3 and Hap3-only cohorts to each other and to themselves. The more similar the two cohorts are, the greater the expectation that a previously reported finding should be replicated in an independent sample from the same population. Hap2∩3 and Hap3-only cohorts were found to be substantially similar (Supporting Figure 4).

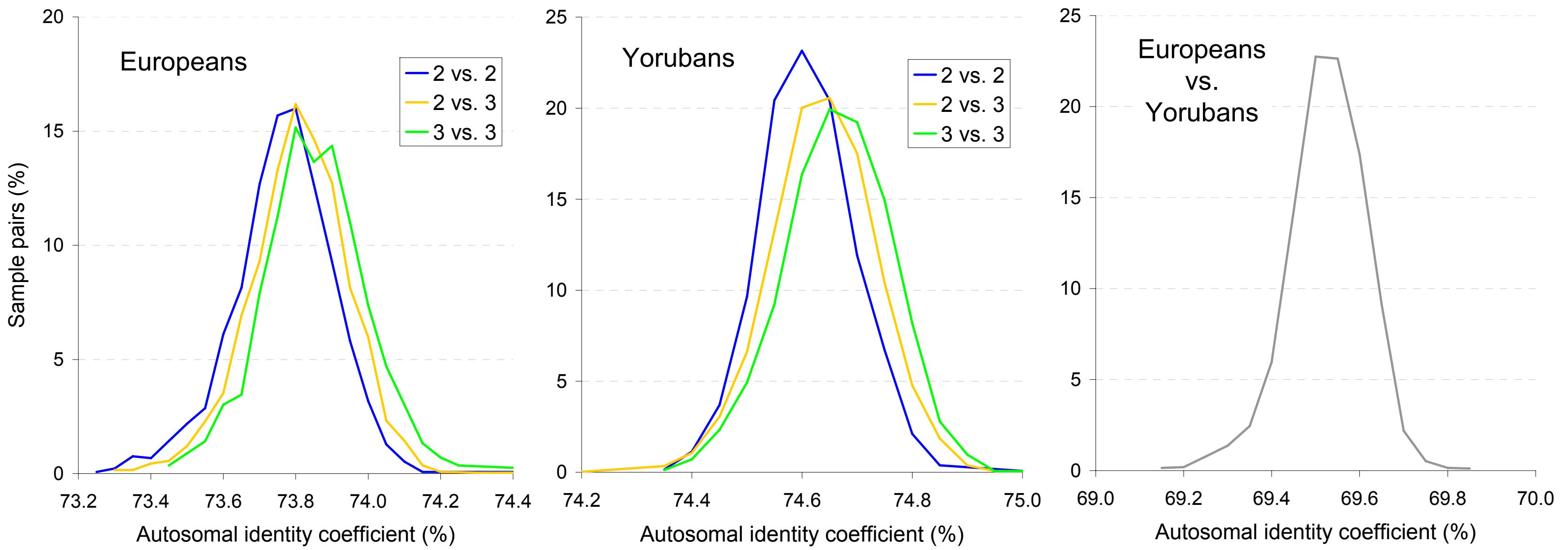

Supporting Figure 4. Correspondence of Hap2∩3 and Hap3-only cohorts. Distributions of pairwise identity coefficients are shown for sample pairs in Phase 3 unphased genotype data. In Europeans (left) and Yorubans (center), samples also genotyped in Phase 2 (2) were separated from those unique to Phase 3 (3). Results are shown separately for intra-cohort (2 vs. 2, 3 vs. 3) and inter-cohort (2 vs. 3) comparisons. Compared to inter-population coefficients (right), inter-cohort differences were slight. Samples without mates were excluded, as were self-self pairs; close relatives are not shown. Identity coefficients were based on all autosomal SNPs with MAF≥1% and het-het=100%.

We also examined the correspondence of MHC identity coefficients for European samples (Supporting Figure 5):

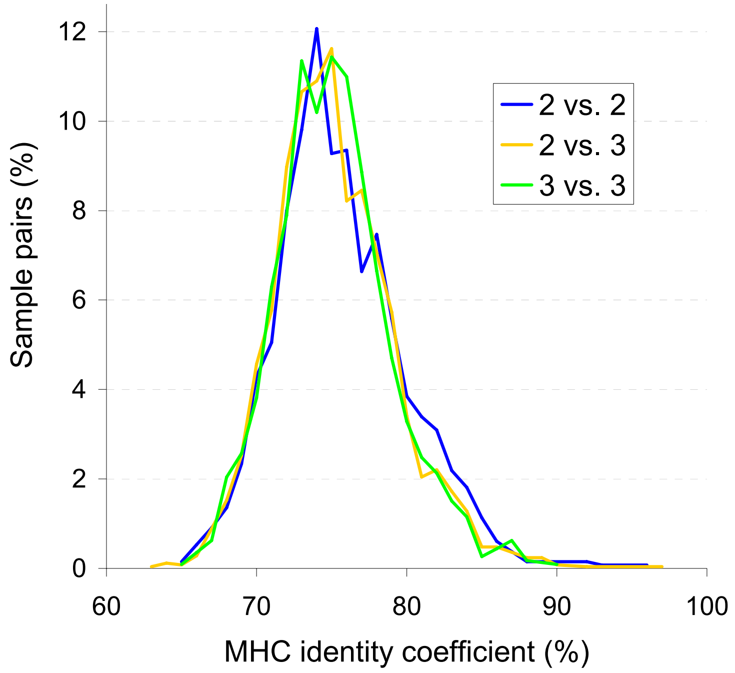

Supporting Figure 5. Similarity of Hap2∩3 and Hap3-only Europeans cohorts at MHC locus. MHC identity coefficients are shown for pairs of Hap3 European samples. See Supporting Figure 4 for details.

These results suggested that the Hap2 and Hap3-only samples were drawn from the same populations, supporting the expectation that results obtained with Hap2 couples would be replicated in Hap3-only couples.
