# Supplementary material for: Absence of Evidence for MHC–Dependent Mate Selection within HapMap Populations
Source: PLoS Genet. 2010 Apr 29;6(4):e1000925. doi: 10.1371/journal.pgen.1000925 (PMC2861700; doi:10.1371/journal.pgen.1000925)
Supplement: Text S4 — Critical impact of an extreme couple on mate-pair MHC dissimilarity. (0.10 MB PDF) [file pgen.1000925.s009.pdf]

Text S4. Critical impact of an extreme couple on mate-pair MHC dissimilarity

Following the replication of results previously reported by Chaix *et al.* in 28 European couples [13], the number of random trials was increased from 1,000 to 100,000, leading to a weaker *P* value (*P* = 0.022) than previously reported (*P* = 0.015) for the MHC dissimilarity of mate pairs relative to random pairs (Table 1). Subsequently removing the single most MHC-dissimilar couple, then repeating the analysis, led to an insignificant result (*P* = 0.052; Table 1 and Supporting Figure 6):

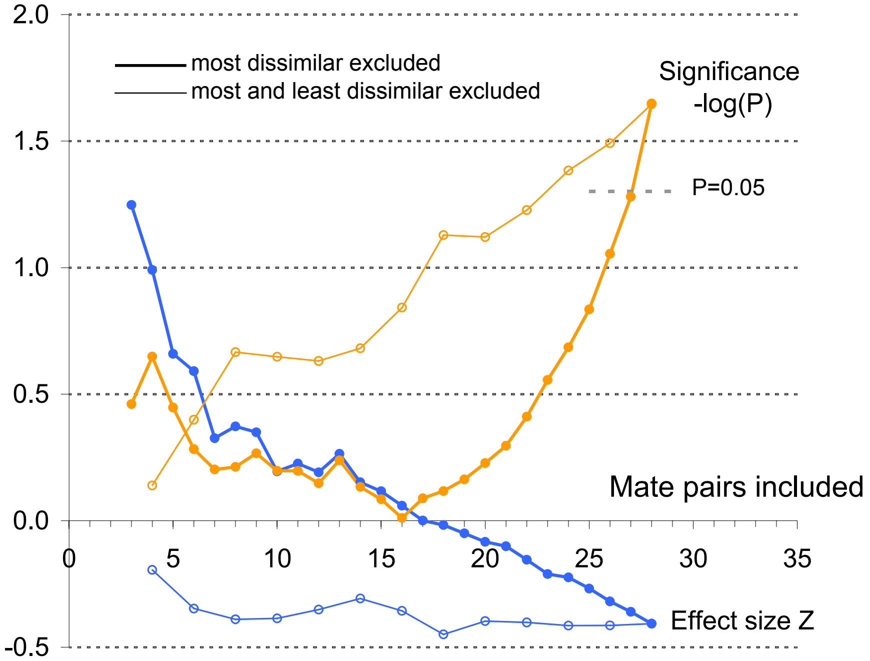

Supporting Figure 6. Impact of extremes on mate-pair MHC dissimilarity in HapMap Phase 2 Europeans. The significance (*P*) and effect size (*Z*) of mate-pair MHC dissimilarity relative to random pairs (for *P*) or non-mate pairs (for *Z*) is shown for analyses conducted with the 28 couples analyzed by Chaix *et al.* [13], using identical data and methods except for 100,000 random trials instead of 1,000. Analyses were repeated successively following the exclusion of the most MHC-dissimilar couple (broad lines), or the simultaneous exclusion of the most and least MHC-dissimilar couples (thin lines).
