# Supplementary material for: Absence of Evidence for MHC–Dependent Mate Selection within HapMap Populations
Source: PLoS Genet. 2010 Apr 29;6(4):e1000925. doi: 10.1371/journal.pgen.1000925 (PMC2861700; doi:10.1371/journal.pgen.1000925)
Supplement: Text S5 — Analysis of Hap2∩3 couples with Hap2 and Hap3 genotypes. (0.12 MB PDF) [file pgen.1000925.s010.pdf]

Text S5. Analysis of Hap2∩3 couples with Hap2 and Hap3 genotypes

Because two sets of SNPs (Hap2 and Hap3) underlie identity coefficients plotted in Figure 2 and Figure 3, coefficients based on Hap3 data for couples present in both phases (2∩3) are shown in Supporting Figure 7:

a. European couples

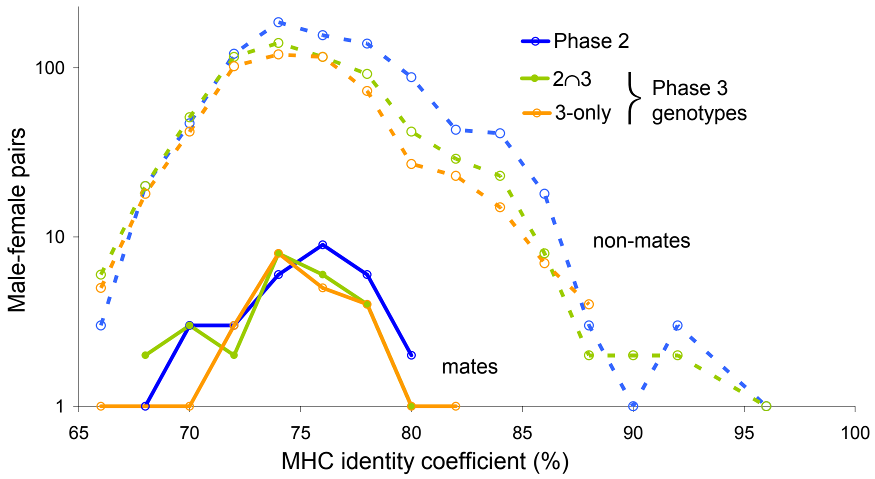

b. Yoruban couples

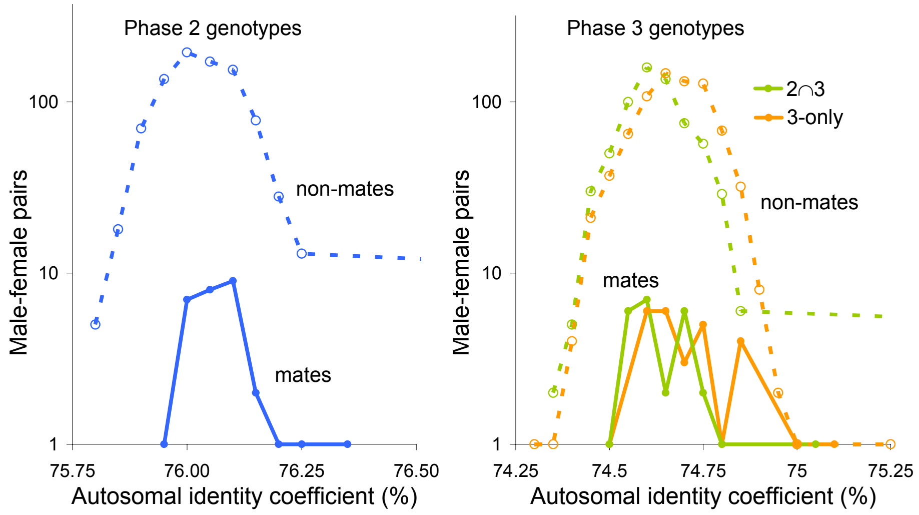

Supporting Figure 7. Identity coefficients of HapMap couples. Distributions of coefficients based on Hap3 genotypes are similar for couples also present in Hap2 (2∩3) and those unique to Hap3 (3-only). Coefficients were based on unphased genotypes, MAF ≥1%, and het-het=100%. Autosomal identity coefficients were lower in Phase 3 because fewer SNPs with low minor allele frequencies were genotyped.
