# Supplementary material for: Absence of Evidence for MHC–Dependent Mate Selection within HapMap Populations
Source: PLoS Genet. 2010 Apr 29;6(4):e1000925. doi: 10.1371/journal.pgen.1000925 (PMC2861700; doi:10.1371/journal.pgen.1000925)
Supplement: Text S6 — Discrepancy detected in Hap2 Yorubans. (0.09 MB PDF) [file pgen.1000925.s011.pdf]

Text S6. Discrepancy detected in Hap2 Yorubans

In Hap2 Yorubans, the mean relatedness was significantly higher among mate pairs than non-mate pairs ($P = 0.004$; Supporting Table 3), as was previously reported [13]. This result held even when only the couples common to Hap2 and Hap3 (2∩3) were examined ($P = 0.011$), and even if only 2∩3 SNPs were used ($P = 0.034$), although significance declined with each step. However, using the very same samples and SNPs, the result became statistically insignificant with Hap3 data ($P \geq 0.092$).

Data	Couples		SNPs	Relatedness				*P*	Z
				Mates		Non-mates			
	Subset	N		mean	s.d.	mean	s.d.		
Hap2	All	27	all	0.00205	0.004	0.00003	0.004	0.00356	0.56
	2∩3	24	all	0.00186	0.004	-0.00012	0.004	0.01088	0.56
			2∩3	0.00145	0.004	-0.00003	0.003	0.03377	0.44
Hap3	2∩3	24	all	0.00109	0.004	-0.00004	0.003	0.12554	0.33
			2∩3	0.00111	0.004	-0.00003	0.003	0.09169	0.35
	3-only	28	all	0.00165	0.005	-0.00005	0.004	0.01648	0.42
			2∩3	0.00178	0.005	-0.00005	0.004	0.00659	0.49

Supporting Table 3. Non-replication of Hap2 results with Hap3 genotypes in Yoruban couples. Overall (autosomal) relatedness is shown for mate and non-mate pairs. Results are a subset of those presented in Table S3, and were calculated with unphased genotypes, MAF≥1% and het-het=100%. *P* values for Hap3-only samples represent one-tailed tests for greater relatedness in mates than expected.

This discrepancy between Hap2 and Hap3 was confirmed with phased genotypes (not shown). It appears that subtle differences between Hap2 and Hap3 genotypes, despite very high concordance for self-self identity (≥ 98.0%; Supporting Figure 3 in Text S3), affect mate-mate identity coefficients slightly more than they affect non-mate coefficients, an effect that we did not observe in Europeans (not shown). It remains unclear whether this discrepancy is random or systematic.
