# Supplementary material for: Absence of Evidence for MHC–Dependent Mate Selection within HapMap Populations
Source: PLoS Genet. 2010 Apr 29;6(4):e1000925. doi: 10.1371/journal.pgen.1000925 (PMC2861700; doi:10.1371/journal.pgen.1000925)
Supplement: Text S7 — Genome-wide relatedness and recombination rate. (0.09 MB PDF) [file pgen.1000925.s012.pdf]

Text S7. Genome-wide relatedness and recombination rate

Supporting Figure 8 summarizes the examination of genome-wide mean relatedness and recombination rate in Hap2 and Hap3 European mates and non-mate pairs, while Supporting Table 4 lists detailed results for Europeans and Yorubans. Results presented in the text for Hap2 are based on phased genotypes and a het-het score of 50% in the interest of the closest comparison with Chaix *et al.* [13], while Hap3 results are based on unphased genotypes and het-het=100% for consistency with the current study. Supporting Table 4 provides results for Hap2 unphased genotypes and het-het=100% to illustrate the minor impact of these differences.

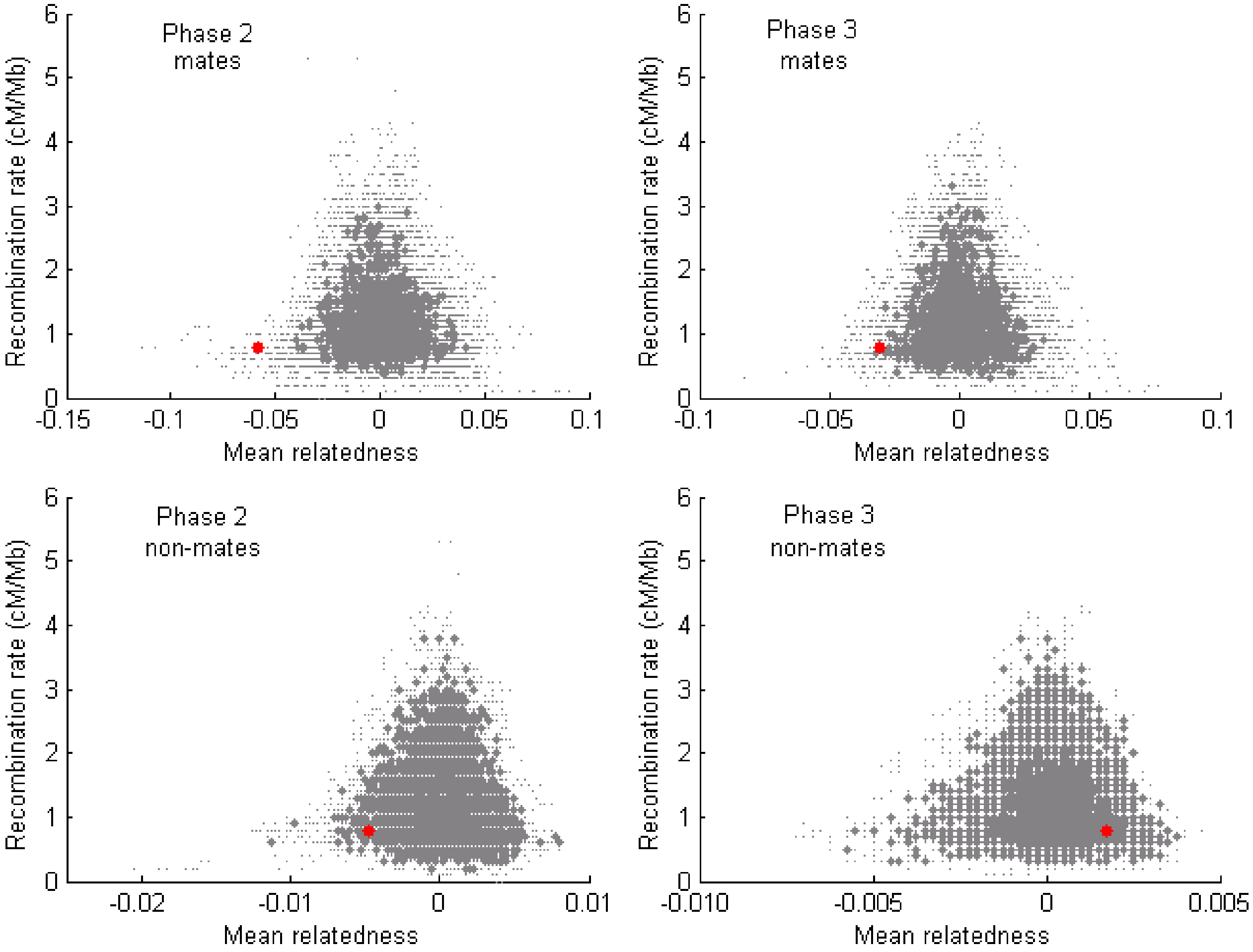

Supporting Figure 8. Genome-wide examination of relatedness and recombination rate in HapMap Europeans. Mean relatedness in either mates or non-mates is plotted against recombination rate for 3.6 Mbp segments throughout the genome (grey; see Methods) and for the MHC locus (red). Note that scales on X-axis differ. Results are shown for unphased genotypes, SNPs with MAF ≥ 1% and a het-het score of 100%. Results were similar with het-het = 50% and, in Phase 2 samples, with phased genotypes (not shown but compare upper left panel with Figure 2A of Chaix *et al.* [13]).

Population	Comparison	Phase	Genotypes	Het-het	Total segments	Mates			Nonmates		
						N	%	Regions	N	%	Regions
Europeans	Relatedness	2	Phased	0.5			0.4	9			
				0.5	8529	82	1.0	18	265	3.1	47
				1.0	8529	50	0.6	9	384	4.5	68
			Unphased	1.0	8472	48	0.6	11	334	3.9	65
		3	Unphased	1.0	8336	133	1.6	34	7583	91.0	53
	Relatedness and recombination	2	Phased	0.5			0.1				
				0.5	2165	62	2.9	16	124	5.7	21
				1.0	2165	36	1.7	8	149	6.9	30
			Unphased	1.0	2139	35	1.6	9	134	6.3	27
		3	Unphased	1.0	2064	73	3.5	19	1753	84.9	135
Yorubans	Relatedness	2	Phased	0.5			91				
				0.5	8538	8265	96.8	46	151	1.8	28
				1.0	8538	8343	97.7	43	75	0.9	21
			Unphased	1.0	8487	8280	97.6	43	76	0.9	21
		3	Unphased	0.5	8397	7940	94.6	49	351	4.2	66
				1.0	8397	7845	93.4	53	150	1.8	36
	Relatedness and recombination	2	Phased	0.5			83				
				0.5	2168	2012	92.8	134	67	3.1	18
				1.0	2168	2048	94.5	132	33	1.5	14
			Unphased	1.0	2147	2030	94.6	131	35	1.6	14
		3	Unphased	1.0	2106	1807	85.8	128	73	3.5	21

Supporting Table 4. Genome-wide examination of relatedness and recombination rate. For 3.6 Mbp segments throughout the genome (see caption of Supporting Figure 8), those with mean relatedness lower than the MHC locus were tallied, separately for mates and for non-mates. Also shown is the number of distinct regions remaining when overlapping segments were joined. The analysis was repeated for segments whose recombination rate was less than or equal to that of the MHC locus. Shading indicates results reported by Chaix *et al.* [13], and cells are left blank for results not previously reported.
